# Effects of Bufei Yishen Granules Combined with Acupoint Sticking Therapy on Pulmonary Surfactant Proteins in Chronic Obstructive Pulmonary Disease Rats

**DOI:** 10.1155/2016/8786235

**Published:** 2016-09-06

**Authors:** Yange Tian, Jiansheng Li, Ya Li, Yuqiong Dong, Fengjia Yao, Jing Mao, Linlin Li, Lili Wang, Shan Luo, Minghang Wang

**Affiliations:** ^1^Collaborative Innovation Center for Respiratory Disease Diagnosis and Treatment & Chinese Medicine Development of Henan Province, Zhengzhou, Henan 450046, China; ^2^Institute for Geriatrics, Henan University of Traditional Chinese Medicine, Zhengzhou, Henan 450046, China; ^3^Institute for Respiratory Diseases, The First Affiliated Hospital, Henan University of Traditional Chinese Medicine, Zhengzhou, Henan 450008, China; ^4^Central Laboratory and Respiratory Pharmacological Laboratory of Chinese Medicine, The First Affiliated Hospital, Henan University of Traditional Chinese Medicine, Zhengzhou, Henan 450008, China

## Abstract

Our previous studies have demonstrated the beneficial effects of Bufei Yishen granules combined with acupoint sticking therapy (the integrated therapy) in chronic obstructive pulmonary disease (COPD), but the underlying mechanism remains unclear. Dysfunction of pulmonary surfactant proteins (SPs, including SP-A, SP-B, SP-C, and SP-D) may be included in pathophysiology of COPD. This study aimed to explore the mechanism of the integrated therapy on SPs. COPD rat models were established. The treatment groups received Bufei Yishen granules or acupoint sticking or their combination. Using aminophylline as a positive control drug. The levels of SPs in serum, BALF, and lung were measured. The results showed that the integrated therapy markedly reduced the levels of SPs in serum and increased these indicators in the lung. The integrated therapy was better than aminophylline in reducing the levels of SPs and was better than Bufei Yishen granules in reducing SP-A, SP-C, and SP-D in serum. The integrated therapy was better than aminophylline and Bufei Yishen granules in increasing SP-A, SP-B, and SP-D mRNA in the lung. SP-A and SP-D in BALF were positively correlated with PEF and EF50. The levels of SPs are associated with airway limitation. The beneficial effects of the integrated therapy may be involved in regulating pulmonary surfactant proteins.

## 1. Introduction

Chronic obstructive pulmonary disease (COPD) has been a human and economic burden worldwide with its high morbidity and mortality [1]. Pulmonary surfactant, synthesized and secreted by alveolar type II epithelial cells (AT-II), is a complex fluid that comprises phospholipids and four proteins (SP-A, SP-B, SP-C, and SP-D) with different biological functions. Dysfunction of pulmonary surfactant proteins may be included in pathophysiology of COPD [2]. SP-A and SP-D regulate surfactant metabolism and have immunologic functions, while SP-B and SP-C play a direct role in the organization of the surfactant structure in the interphase and in the stabilization of the lipid layers during the respiratory cycle [3]. Pulmonary surfactant proteins have important function on reducing the alveolar surface tension and preventing alveolar collapse and the airway walls collapse [4].

In treatment, pharmacologic therapies (bronchodilators, corticosteroids, phosphodiesterase-4 inhibitors, and *β*2-agonists) and nonpharmacologic therapies (pulmonary rehabilitation and smoking cessation) are frequently used to manage COPD as recommended by the World Health Authority (WHO) and GOLD. Although these therapies have proved to reduce exacerbations and alleviate symptoms, there is little evidence to suggest that they can suppress the progression of this disease. Traditional Chinese Medicine (TCM), including internal and external therapies, has attracted more attention in recent years because of its potential advantages in improving symptoms, reducing the frequency of acute exacerbation, and improving quality of life in stable COPD [5]. In TCM, COPD belongs to the category of lung distention (Feizhang disease), and the pattern of lung-kidney qi deficiency is one of the most common syndromes in the patients in stable phase [6]. Bufei Yishen granules (patent: ZL.201110117578.1), a special oral prescription for lung-kidney qi deficiency syndrome, were clinically proved effective in relieving clinical symptoms and reducing the frequency of acute exacerbations in stable COPD patients [7]. Additionally, Bufei Yishen granules were also confirmed effective in ameliorating systemic and airway inflammation and remodeling in a cigarette smoke/bacterial exposure induced COPD rat model [8, 9]. Shu-Fei Tie (patent: ZL.200810049332.3) is a popular clinically used ointment for acupoint sticking in external therapy which can excite vital qi in the human body and has proved satisfactory therapeutic effect in COPD treatment with its high safety, convenience, and fewer side effects [10, 11]. In our previous study, Bufei Yishen granules combined with Shu-Fei Tie has shown the beneficial effects in terms of the frequency of acute exacerbation, lung function, clinical symptoms, six-minute walking distance, dyspnea grade, and quality of life, over a 4-month treatment period and 6 months of follow-up in patients with stable COPD [12]. Our previous animal experimental study has also shown that this approach improves pulmonary function and lung pathological impairment in COPD rats [13].

However, the mechanism of Bufei Yishen granules combined with Shu-Fei Tie remains unclear. Thus, our current study was performed to explore the mechanism of Bufei Yishen granules combined with Shu-Fei Tie therapy on pulmonary surfactant proteins.

## 2. Materials and Methods

### 2.1. Animal

72 Sprague-Dawley rats (equal number of males and females, 2 months old, weighing 180–220 g) were obtained from the Laboratory Animal Center of Henan Province (Special Pathogen Free, SCXK [Henan] 2010-0002) and randomly assigned to the Control, Model, Bufei Yishen (BY), acupoint sticking (AS), Bufei Yishen + acupoint sticking (BY + AS), and aminophylline (APL) groups, with 12 animals in each group. Experimental protocols were approved by the Experimental Animal Care and Ethics Committees of the First Affiliated Hospital at Henan University of Traditional Chinese Medicine, Zhengzhou, China.

### 2.2. Cigarette and Bacteria

Commercial cigarettes (Hongqiqu® filter cigarette, Henan, China) were provided by Henan Tobacco Industry Co., Ltd., and each of these cigarettes contained 1.0 mg nicotine, 11 mg CO, and 10 mg tar oil, according to the manufacturer's specifications. Klebsiella pneumoniae (strain: 46114) was purchased from the National Center For Medical Culture Collection (Beijing, China) and prepared at a concentration of 6 × 10^8^ colony forming units (CFU) per milliliter before being administered to the animals.

### 2.3. Drugs

(1) Aminophylline tablets (Xinhua, Shandong, China, 0.1 g/tablet) were crushed prior to administration to the animals. (2) Bufei Yishen granules [Ren Shen (Ginseng Radix et Rhizoma) 9 g, Huang Qi (Astragali Radix) 15 g, Shan Zhu Yu (Corni Fructus) 12 g, Yin Yang Huo (Epimedii Herba) 9 g, Gou Qi Zi (Lycii Fructus) 12 g, Wu Wei Zi (Schisandrae Chinensis Fructus) 9 g, Zhe Bei Mu (Fritillariae Thunbergii) 9 g, Zi Su Zi (*Perilla frutescens*) 9 g, Chen Pi (Citri Reticulatae Pericarpium) 9 g, Chi Shao (Paeoniae Rubra Radix) 9 g, Ai Di Cha (Herba Ardisiae Japonicae) 15 g, Di Long (*Lumbricus*) 12 g] were prepared by the Department of Pharmacology in the First Affiliated Hospital of Henan University of Traditional Chinese Medicine, Zhengzhou, China. (3) Shu-Fei Tie mainly consisted of Bai Jie Zi (Semen Brassicae) 10 g, Yan Hu Suo (Rhizoma Corydalis) 5 g, Xi Xin (*Asarum heterotropoides*) 5 g, Yuan Hua (*Daphne genkwa*) 10 g, and Gan Jiang (Zingiberis Rhizoma) 5 g (3.0 g/tube). The main component of Shu-Fei Tie placebo was Carbopol, diatomaceous earth, and glycerine, each unit equivalent to 3.0 g. The appearance and odor of Shu-Fei Tie placebo were similar to Shu-Fei Tie. Shu-Fei Tie and its placebo were produced and packed by the Department of Pharmacology in the First Affiliated Hospital of Henan University of TCM, which was the reform base of TCM preparation and dosage formulation.

### 2.4. COPD Rat Model Preparation

Rats were housed in individual ventilated cages (Fengshi, Suzhou, China) seven days before the experiment and were provided free access to sterile food and water. After accommodating to the facility for 7 days, COPD rats were exposed to cigarette smoke and* Klebsiella pneumoniae* (KP) for model establishment according to previously described methodology [14]. Animals were exposed to smoke (smoke concentrations, 3,000 ± 500 ppm) for 30 min, twice a day for 12 weeks.* Klebsiella pneumoniae* solution (0.1 mL, 6 × 10^8^ colony forming units/mL) was dropped into the two nostrils in an alternate fashion, once every 5 days, for the first 8 weeks. The successful generation of a COPD rat model was evaluated according to symptoms, lung function, and pulmonary pathology [15].

### 2.5. Administration

From week 9 through 20, rats in the Control and Model were intragastrically given normal saline (2 mL/animal, b.i.d) and Shu-Fei Tie placebo (2 times/week); Bufei Yishen granules (4.44 g/kg·d, b.i.d) and Shu-Fei Tie placebo were given to the BY; normal saline and Shu-Fei Tie were given to the AS; Bufei Yishen granules (4.44 g/kg·d, b.i.d) and Shu-Fei Tie were given to the BY + AS; and aminophylline (2.3 mg/kg·d, b.i.d) and Shu-Fei Tie placebo were given to the APL. Dosage adjustments were made weekly according to body mass. The equivalent dosages were calculated by using the following formula: *D*_rat_ = *D*_human_ × (*I*_rat_/*I*_human_)×(*W*_rat_/*W*_human_)^2/3^, where *D* is dose, *I* body shape index, and *W* body weight. Rats in each group were sacrificed at week 20.

Methods of acupoint sticking are as follows. The acupoint sticking was applied at Dazhui, Feishu (both sides), and Shenshu (both sides). The method of acupoint sticking and skin injury treatment was according to [13]. After the rats were mildly anesthetized, the hair of the acupuncture point was removed. Shu-Fei Tie ointment (0.1 g/point) or Shu-Fei Tie placebo (0.1 g/point) was placed on the acupoints and covered with medical adhesive tape; administration was performed twice each week (Monday and Thursday in this study). Each treatment lasted for 4–6 h. The rats were sacrificed at the end of the 20th week and peripheral blood, bronchoalveolar lavage fluid (BALF), and lung tissue were collected for indexes measurement.

### 2.6. Pulmonary Function Tests

Tidal volume (*V*_*T*_), peak expiratory flow (PEF), and expiratory flow at 50% tidal volume (EF50) were measured by an unrestrained whole body plethysmograph (Buxco, NC, USA) at weeks 0, 8, and 20.

### 2.7. Enzyme-Linked Immunosorbent Assay

SP-A, SP-B, SP-C, and SP-D concentrations in BALF and serum were quantified by using a commercial ELISA kit (RapidBio, USA) according to the manufacturer's protocol.

### 2.8. Quantitative Real-Time PCR Analysis

SP-A, SP-B, SP-C, and SP-D mRNA were measured by quantitative real-time PCR. Total RNA was extracted by using TRIzol reagent (Life Technologies, NY, USA) according to the manufacturer's instructions. Reverse transcription (RT) was performed by using Supre® III First-Strand Synthesis Super Mix for qRT-PCR Kit (Life Technologies, NY, USA), and real-time PCR reactions were performed by using Platinum SYBR® Green® Super Mix-UDG Kit (Life Technologies, NY, USA) on an ABI 7300 real-time instrument (ABI, CA, USA). The cycling conditions involved an enzyme activation step at 95°C for 2 min, followed by 40 cycles of 95°C for 15 s and 60°C for 30 s. At the end of the PCR, to evaluate specific amplification of the target genes, melting curves ranging from 60 to 95°C were also included in each run. Primers for SP-A, SP-B, SP-C, and SP-D were designed and synthesized by Generay Biotech Co. Ltd. (Shanghai, China), and the sequences used in this study are shown in Table 1.

## 3. Results

### 3.1. Pulmonary Function

**Figure 1:**
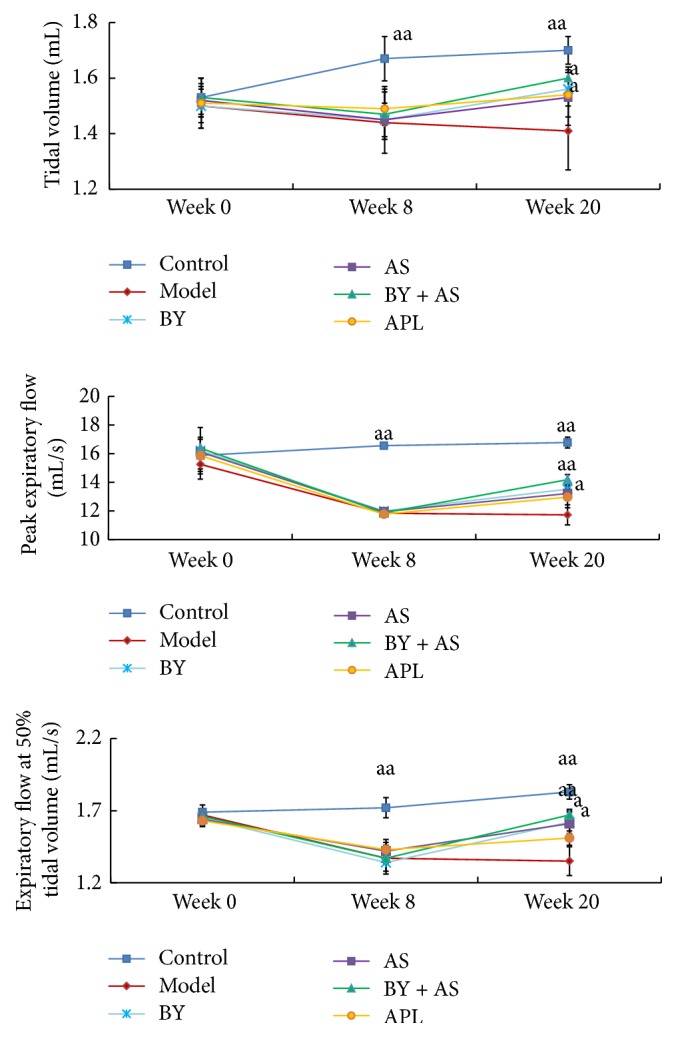
Measurement of lung function (*V*_*T*_, PEF, and EF50) in each group at weeks 0, 8, and 20. Control: control group; Model: model group; BY: Bufei Yishen group; AS: acupoint sticking group; BY + AS: Bufei Yishen + acupoint sticking group; APL: aminophylline group. *N* = 10. Values represent the mean ± SEM. ^aa^ *P* < 0.01, ^a^ *P* < 0.05 versus Model.

As shown in Figure 1, at the 8th week, *V*_*T*_, PEF, and EF50 in the Model were decreased compared with those in Control (*P* < 0.01). At the 20th week, *V*_*T*_, PEF, and EF50 in BY and BY + AS were increased compared with those in Model (*P* < 0.01 or *P* < 0.05), while only *V*_*T*_ in APL and EF50 in AS were increased compared with Model (*P* < 0.05). There was no significant difference among groups (*P* > 0.05).

### 3.2. SP-A, SP-B, SP-C, and SP-D Concentration in Serum

**Figure 2:**
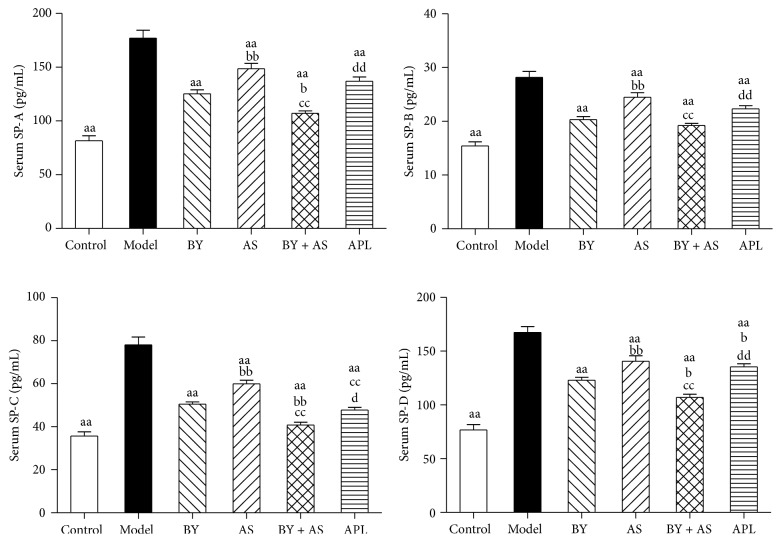
The levels of SP-A, SP-B, SP-C, and SP-D in serum in all treatment group. Control: control group; Model: model group; AS: acupoint sticking group; BY: Bufei Yishen group; BY + AS: Bufei Yishen + acupoint sticking group; APL: aminophylline group. Values represent the mean ± SEM. ^aa^ *P* < 0.01 versus Model; ^bb^ *P* < 0.01, ^b^ *P* < 0.05 versus BY; ^cc^ *P* < 0.01 versus AS; ^dd^ *P* < 0.01, ^d^ *P* < 0.05 versus BY + AS.

As shown in Figure 2, compared with Control, concentration of SP-A, SP-B, SP-C, and SP-D was increased significantly in Model (*P* < 0.01). Compared with Model, the indexes above were decreased in all treatment groups (*P* < 0.01). Compared with APL, the levels of SP-A, SP-B, SP-C, and SP-D in BY + AS were decreased (*P* < 0.01 or *P* < 0.05) and the level of SP-D in BY was decreased (*P* < 0.05). Compared with acupoint sticking group, concentration of SP-A, SP-B, SP-C, and SP-D in BY and BY + AS was decreased (*P* < 0.01), and the level of SP-C in APL was decreased (*P* < 0.01). Compared with Bufei Yishen, the levels of SP-A, SP-C, and SP-D in BY + AS were decreased (*P* < 0.01 or *P* < 0.05).

### 3.3. SP-A, SP-B, SP-C, and SP-D Concentration in BALF

**Figure 3:**
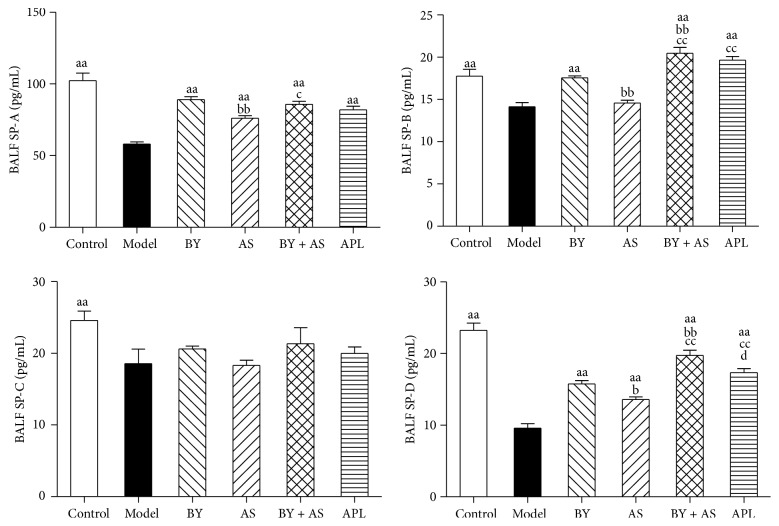
The levels of SP-A, SP-B, SP-C, and SP-D in BALF in all treatment groups. Control: control group; Model: model group; AS: acupoint sticking group; BY: Bufei Yishen group; BY + AS: Bufei Yishen + acupoint sticking group; APL: aminophylline group. Values represent the mean ± SEM. ^aa^ *P* < 0.01 versus Model; ^bb^ *P* < 0.01, ^b^ *P* < 0.05 versus BY; ^cc^ *P* < 0.01, ^c^ *P* < 0.05 versus AS; ^d^ *P* < 0.05 versus BY + AS.

As shown in Figure 3, compared with Control, concentration of SP-A, SP-B, SP-C, and SP-D was decreased significantly in Model (*P* < 0.01). Compared with Model, concentration of SP-A and SP-D in all treatment groups was increased (*P* < 0.01), and SP-B concentration in BY, APL, and BY + AS was increased (*P* < 0.01). Compared with APL, the level of SP-D in BY + AS was increased (*P* < 0.05). Compared with AS, the levels of SP-A, SP-B, and SP-D in BY and BY + AS were increased (*P* < 0.01 or *P* < 0.05), and the levels of SP-B and SP-D in APL were increased (*P* < 0.01). Compared with BY, the levels of SP-B and SP-D in BY + AS were increased (*P* < 0.01).

### 3.4. SP-A, SP-B, SP-C, and SP-D mRNA in Lung Tissue

**Figure 4:**
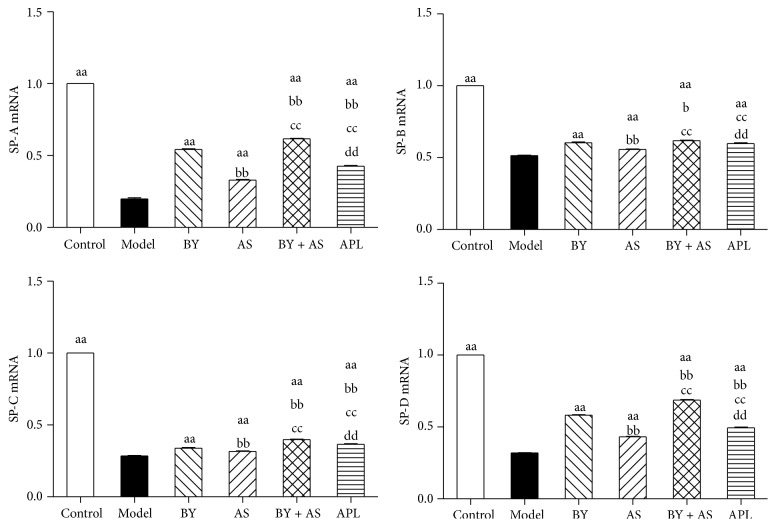
SP-A, SP-B, SP-C, and SP-D mRNA in the lung in all treatment groups. Control: control group; Model: model group; AS: acupoint sticking group; BY: Bufei Yishen group; BY + AS: Bufei Yishen + acupoint sticking group; APL: aminophylline group. Values represent the mean ± SEM. ^aa^ *P* < 0.01 versus Model; ^bb^ *P* < 0.01, ^b^ *P* < 0.05 versus BY; ^cc^ *P* < 0.01 versus AS; ^dd^ *P* < 0.01 versus BY + AS.

As shown in Figure 4, compared with Control, SP-A, SP-B, SP-C, and SP-D mRNA was decreased significantly in Model (*P* < 0.01). Compared with Model, the indexes above were increased in all treatment groups (*P* < 0.01). Compared with aminophylline group, SP-A, SP-B, and SP-D mRNA in BY and BY + AS was increased (*P* < 0.01). Compared with acupoint sticking group, SP-A, SP-B, SP-C, and SP-D mRNA in BY and BY + AS was increased (*P* < 0.01). Compared with BY, SP-A, SP-B, SP-C, and SP-D mRNA in BY + AS was increased (*P* < 0.01).

### 3.5. The Relationship between SP-A, SP-B, SP-C, and SP-D in BALF and Pulmonary Function (PEF and EF50)

**Figure 5:**
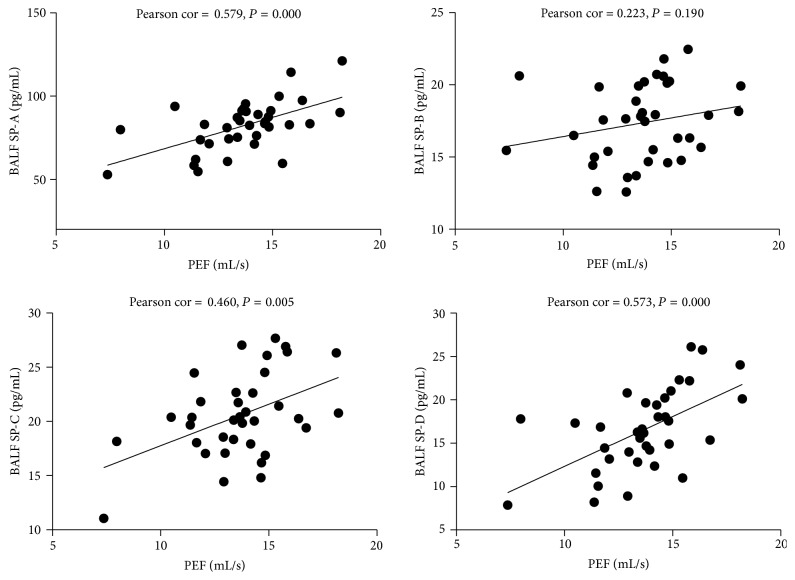
The relationship between SP-A, SP-B, SP-C, and SP-D in BALF and PEF. Significant relationship is noted between BALF SP-A, SP-C, SP-D, and PEF.

**Figure 6:**
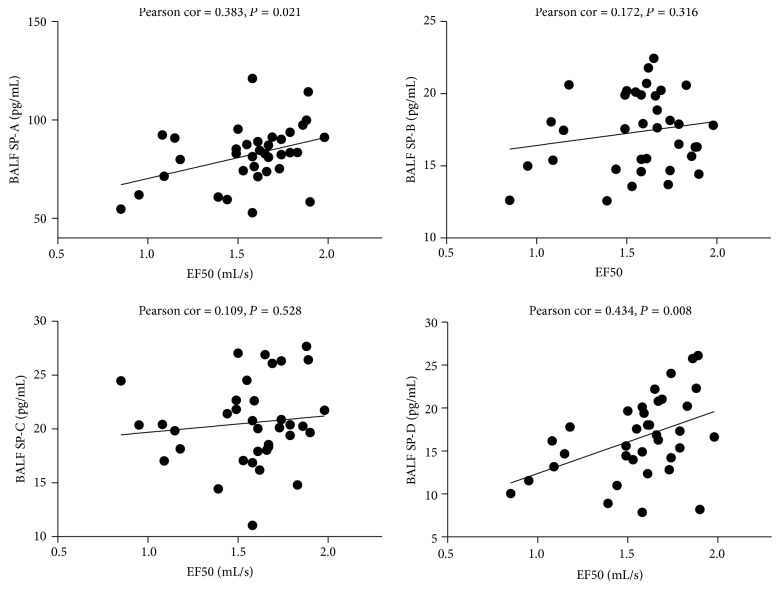
The relationship between SP-A, SP-B, SP-C, and SP-D in BALF and EF50. Significant relationship is noted between BALF SP-A, SP-D, and EF50.

AS shown in Figures 5 and 6, SP-A, SP-C, and SP-D in BALF were significantly positively correlated with PEF (Pearson cor. = 0.579, 0.460, 0.573; *P* = 0.000, 0.005, 0.000); SP-A and SP-D in BALF were significantly positively correlated with EF50 (Pearson cor. = 0.383, 0.434; *P* = 0.021, 0.008).

## 4. Discussion

COPD is one of the major diseases which threaten the human health. Recently, the good curative effects of Traditional Chinese Medicine (TCM), including internal treatment and external therapies, are attracting more and more attention in treatment of COPD. Bufei Yishen granules, a special prescription for lung-kidney qi deficiency syndrome with confirmed curative effects, and its potential targets have been discussed by using systems pharmacology [16]. Shu-Fei Tie, an ointment for acupoint sticking in external therapy, is popularly used in COPD patients with certain therapeutic effects [10, 11]. In our previous clinical and animal studies, Bufei Yishen granules combined with Shu-Fei Tie have been demonstrated to be beneficial in treating stable COPD; however, the mechanism responsible for these effects remains unclear.

The function of pulmonary surfactant proteins is closely related to the generation and progression of COPD. Lin et al. found that SP-A in the exhaled breath condensate (EBC) were significantly decreased in patients with COPD compared with the non-COPD subjects and decreased expression levels of SP-A in EBC were associated with a higher degree of airway limitation [17]. Decreased levels of SP-A may lead to impaired host defense functions of surfactant in the airways, contributing to increased susceptibility to COPD exacerbations [18]. SP-B variants are associated with COPD susceptibility and lung function in COPD patients [19]. A report found that the severity of emphysema was positively associated with the levels in serum SP-D in COPD and increased levels in serum SP-D are associated with the development of COPD [20]. Serum SP-D level is associated with lung function or health status in patients with severe COPD [21]. Thus, SP-D has been confirmed a biological marker of COPD [22, 23].

Our study showed that the levels of SP-A, SP-B, SP-C, and SP-D were increased significantly in serum in COPD rats. All four treatment protocols (Bufei Yishen granules, Shu-Fei Tie, and Bufei Yishen granules combined with Shu-Fei Tie and aminophylline) reduced the indicators above. Bufei Yishen granules combined with Shu-Fei Tie were better than aminophylline in reducing levels of SP-A, SP-B, SP-C, and SP-D. Bufei Yishen granules were better than aminophylline in reducing level of SP-D. Bufei Yishen granules combined with Shu-Fei Tie were better than Bufei Yishen granules in reducing SP-A, SP-C, and SP-D. We also measured SP-A, SP-B, SP-C, and SP-D mRNA in lung of COPD rats. SP-A, SP-B, SP-C, and SP-D mRNA was decreased significantly in COPD rats. All four treatment protocols can increase the expression of the indicators above. Bufei Yishen granules and Bufei Yishen granules combined with Shu-Fei Tie were better than aminophylline in increasing SP-A, SP-B, and SP-D mRNA. Bufei Yishen granules combined with Shu-Fei Tie were better than Bufei Yishen granules in increasing SP-A, SP-B, SP-C, and SP-D mRNA.

Chronic airway limitation is a key pathogenesis of COPD and the lung function is crucial to diagnose and assess the severity of COPD. Tidal volume (*V*_*T*_), peak expiratory flow (PEF), and expiratory flow at 50% tidal volume (EF50) can be obtained easily by using unrestrained whole body plethysmography and reflect the extent of airway limitation. Based on our results, the level of *V*_*T*_, PEF, and EF50 significantly decreased in COPD rats. Bufei Yishen granules and the combination treatment can improve these indexes, while Shu-Fei Tie only improved EF50 and aminophylline only improved *V*_*T*_.

Several reports found that the levels of pulmonary surfactant proteins are associated with airway limitation. The pro-SP-B concentrations in lung of COPD patients are positively associated with severity of airflow limitation as measured by FEV1 % of predicted and FEV1/FVC ratio [24].SP-D levels in BALF are decreased in smokers and patients with COPD and are positively correlated with FEV1/FVC [25]. This study found that SP-A, SP-C, and SP-D in BALF were significantly positively correlated with PEF. SP-A and SP-D in BALF were positively correlated with EF50.

In conclusion, our results indicate that all four treatment protocols can reduce the expression of pulmonary surfactant proteins in serum and increase these indicators in BALF and lung and Bufei Yishen granules combined with Shu-Fei Tie are better than other protocols. The levels of pulmonary surfactant proteins are associated with airway limitation. The beneficial effects of Bufei Yishen granules combined with Shu-Fei Tie may be involved in regulating pulmonary surfactant proteins.

## Figures and Tables

**Table 1:** Primer sequence of SP-A, SP-B, SP-C, and SP-D mRNA.

Gene	Primer	Sequence (5′ → 3′)
GADPH	FW	ACA GCA ACA GGG TGG TGG AC
	RV	TTT GAG GGT GCA GCG AAC TT

SP-A	FW	CTT CAC CCT CTT CTT GAC TGT TG
	RV	TCT CCC TTG TCT CCA CGT CCT

SP-B	FW	TGG CTA CTG CTC CTT CCT ACA CT
	RV	GCG TCT TCC TTG GTC ATC TTTG

SP-C	FW	TGG TCC TCA TCG TCG TGG TGA TTG
	RV	CCT GCA GAG AGC ATT CCA TCT GGA AG

SP-D	FW	ACT CAT CAC AGC CCA CAA CA
	RV	TCA GAA CTC ACA GAT AAC AAG

FW: forward; RV: reverse.
